# Antimicrobial effect and mechanism of bovine lactoferrin against the potato common scab pathogen *Streptomyces scabiei*

**DOI:** 10.1371/journal.pone.0264094

**Published:** 2022-02-25

**Authors:** Masayuki Nakamura, Naoaki Tsuda, Takeshi Miyata, Makoto Ikenaga

**Affiliations:** Faculty of Agriculture, Kagoshima University, Kagoshima, Japan; Okayama University, JAPAN

## Abstract

Lactoferrin (LF) is a multifunctional protein with a broad spectrum of antimicrobial activities. In this study, we investigated the antimicrobial activity of LF against the potato common scab pathogen *Streptomyces scabiei*, which causes severe damage to potato tubers. LF derived from bovine (bLF) had much higher activity against *S*. *scabiei* than human LF. The minimal inhibitory concentration of bLF was 3.9 μM. The effects of both apo-bLF (iron-free) and holo-bLF (iron-saturated) on *S*. *scabiei* were not different. Bovine lactoferricin (LFcinB), a short peptide with a length of 25 amino acid residues located in the N-terminal region of bLF, showed antimicrobial activity against *S*. *scabiei*, similar to that of bLF. These results indicated that the antimicrobial activity of bLF against *S*. *scabiei* cannot be attributed to its iron-chelating effect but to the bioactivity of its peptides. When *S*. *scabiei* was treated with the fusion protein of mCherry-LFcinB (red fluorescent protein) expressed in *Escherichia coli*, the pseudohyphal cells instantly glowed, indicating that the peptide electrostatically binds to the surface of *S*. *scabiei*. An assay of synthetic peptides, with modified number of arginine (Arg) and tryptophan (Trp) residues based on the antimicrobial center (RRWQWR) of LFcinB showed that Trp residues are implicated in the antimicrobial activity against *S*. *scabiei*; however, Arg residues are also necessary to carry Trp residues to the cell surface to fully exert its activity. Although the single amino acid effect of Trp had low activity, Trp derivatives showed much higher activity against *S*. *scabiei*, suggesting that the derivatives effectively bind to the cell surface (cell membrane) by themselves without a carrier. Thus, amino acid derivatives might be considered effective and alternative antimicrobial substances.

## Introduction

Lactoferrin (LF), a member of the transferrin family, is a multifunctional glycoprotein present in milk, sudor, lacrima, saliva, and blood. LF has various properties, including antioxidant, anticancer, and wound healing [1–3]. In particular, it performs a wide range of antimicrobial activities, thereby, playing a critical role in protecting newborn infants from infection [4]. To date, the antimicrobial activity of LF has been studied mainly against human pathogens, including viruses, bacteria, and fungi [5–10]. Recently, the antiviral activity of LF against the novel coronavirus SARS-Cov2 (COVID-19) has also been intensely studied [11–13]. Indeed, LF has a wide spectrum of activities against many types of pathogens, including plant pathogens, such as *Fusarium graminearum* (fungus) [14], *Pseudomonas syringae* (bacterium) [15], and Tobacco mosaic virus [16]. However, the antimicrobial effect of LF on actinomycetes has not been determined in both medical and plant pathology. On the contrary, bifidobacteria belonging to actinomycetes have been reported to exploit LF for effective growth [17]; however, the effect of LF on plant pathogens belonging to actinomycetes is unclear. Therefore, in this study, we investigated the effect of LF on the potato common scab pathogen *Streptomyce*s *scabiei*, an actinomycete, which causes an economically important disease in potato tubers worldwide and is difficult to control in the field [18, 19].

The mechanisms underlying the antimicrobial action of LF are related to its ability to strongly bind to iron [20] or to the direct killing effect of some bioactive peptides present in it [21, 22]. Siderophore-mediated iron acquisition is critical for the successful infection of hosts by both human and plant microbial pathogens [23]. In some microbial pathogens, growth is inhibited by the ability of LF to sequester iron [24]. LF harbors antimicrobial peptides, such as LF1-11 [25], lactoferampin [26], and lactoferricin [27]. These peptides are rich in hydrophobic and cationic amino acids, which are thought to be important for antimicrobial effects [28–30]. In this study, we focused on bovine lactoferricin (LFcinB) (S1 Fig), which is well studied, and sought to understand the contribution of the core motif (RRWQWR) [31–33] situated in the peptide to the antimicrobial activity against *S*. *scabiei*.

This study is the first to report the antimicrobial effect of LF on *S*. *scabiei* and to clarify the mechanism underlying its action. We also examined the potential of amino acid derivatives as alternative antimicrobial agents to control *S*. *scabiei* from the perspective of LF properties.

## Materials and methods

### Bacterial strains and culture

*S*. *scabiei* S58, isolated from potatoes grown in Kagoshima, Japan [34], was used in this study. The S58 isolate was cultured at 28 °C for 7 days on starch agar (STA) (1% starch, 0.1% sucrose, 0.1% yeast extract, 0.01% NaNO_3_, 0.01% MgSO_4_·7H_2_O, 0.01% KH_2_PO_4_, 0.01% KCl, 1.5% agar) to obtain spores. For antimicrobial assays, the spores of the isolate were incubated at 28 °C in tryptic soy broth (TSB) (Becton Dickinson, Sparks, USA). *Escherichia coli* strains JM109 and BL21(λDE3) were grown at 37 °C in Luria-Bertani broth supplemented with 100 μg/mL ampicillin for propagation.

### Preparation of antimicrobial test substances

Human-derived LF (hLF) and bovine-derived LF (bLF) were purchased from FUJIFILM Wako Pure Chemical Corporation, Osaka, Japan. Iron-depleted (apo) bLF and iron-saturated (holo) bLF were prepared using the method described by Wang et al. (2013) [35]. The iron content of apo- and holo-LFs was confirmed using an FeC-test kit (FUJIFILM Wako); considering the theoretical value of 2 M iron per 1 M LF as 100% saturation, the saturation of apo- and holo-LFs was determined to be 0.35% and 95.5%, respectively. For bLF hydrolysis, 50 mg/mL bLF was hydrolyzed in 3% (w/w) porcine pepsin (Merck/Sigma-Aldrich, St. Louis, MO, USA) according to the procedure described by Tomita et al. (1991) [36]. LFcinB (purity ≥95%) was purchased from Merck/Sigma-Aldrich. Synthetic peptides (RRWQWR, RRWQRR, RRRQRR, RRWWWR, and QQWWWQ) were also manufactured by Merck/Sigma-Aldrich. Amino acid derivatives were purchased from Tokyo Chemical Industry Co., Ltd. (Tokyo, Japan). All the synthetic peptides were easily soluble in water.

### Antimicrobial assays

A 12-well plate was used to assess the antimicrobial activity of LF, hydrolysate, LFcinB, and synthetic peptides. One milliliter of TSB containing each test substance was added to each well and then the spores of *S*. *scabiei* S58, prepared as described above, were added into each well at a final concentration of 2 × 10^5^ spores/mL. The mixture was incubated at 28 °C for 72 h, and the absorbance was measured at 600 nm to monitor bacterial growth. To further confirm whether the antimicrobial activity is due to growth inhibition or bactericidal effect, 1 mL of TSB containing 2 ×10^3^ spores and bLF (1 mg/mL) was incubated at 28 °C and directly cultured on STA after 5, 10, 15, 24, 48, and 72 h of incubation to count the emerging colonies. All assays were performed in duplicate and were repeated three times.

### Protein expression in *E*. *coli*

The gene fragment encoding LFcinB was constructed by overlap extension (OE)-PCR using the deoxyoligonucleotides LFcinB-FW (5′-TTCAAATGCCGCCGTTGGCAGTGGCGTATGAAAAAACTGGGTG-3′) and LFcinB-RV (5′-GAACGCGCGACGCACGCAGGTAATAGACGGCGCACCCAGTTT-3′), the codon usage of which was optimized for expression in *E*. *coli*. These two deoxyoligonucleotides have a ten-base complementary overlap at their 3′-end. The gene encoding mCherry (a red fluorescent protein) was amplified by pmCherry (Takara Bio, Otsu, Japan) using primers mCherry-pET23-FW (5′-AAGGAGATATA*CATATG*GTGAGCAAGGGCGAGGAGGA-3′)/mCherry-pET23-RV (5′-GGTGGTGGTG*CTCGAG*CTTGTACAGCTCGTCCATGC-3′). The gene encoding the fusion protein mCherry-LFcinB was constructed by OE-PCR. First, the two fragments were amplified from the above mCherry and LFcinB fragments using primers mCherry-pET23-FW/mCherry-LFcinB-B (5′-ACGGCGGCATTTGAA CTTGTACAGCTCGTCCATGC-3′), and mCherry-LFcinB-C (5′- GACGAGCTGTACAAGTTCAAATGCCGCCGTTGGCA-3′)/LFcinB-pET23-RV (5′-GGTGGTGGTG*CTCGAG*GAACGCGCGACGCACGCAGG-3′), and the two newly obtained fragments were joined with mCherry-pET23-FW/LFcinB-pET23-RV. The nucleotides in italics in the above primer sequences indicate restriction enzyme sites. PrimeSTAR GXL DNA polymerase (Takara Bio) was used for DNA amplification. The amplified fragments were cloned into pET-23b (Merck/Novagen, Madison, WI, USA), digested with NdeI and XhoI, via an In-Fusion HD Cloning kit (Takara Bio). The expression vectors obtained were transferred into *E*. *coli* BL21 (λDE3) cells (Merck/Novagen) via electroporation. Protein expression was induced with 1 mM isopropyl β-D-1-thiogalactopyranoside and incubated for 12 h at a low temperature (15 °C) to reduce its toxicity to the cells. The expressed proteins were purified using a Capturem His-Tagged Purification kit (Takara Bio) and adjusted to a concentration of 75 μg/mL before treatment against the pseudohyphal cells of *S*. *scabiei* S58.

## Results

### Antimicrobial activity of LF

**Fig 1:**
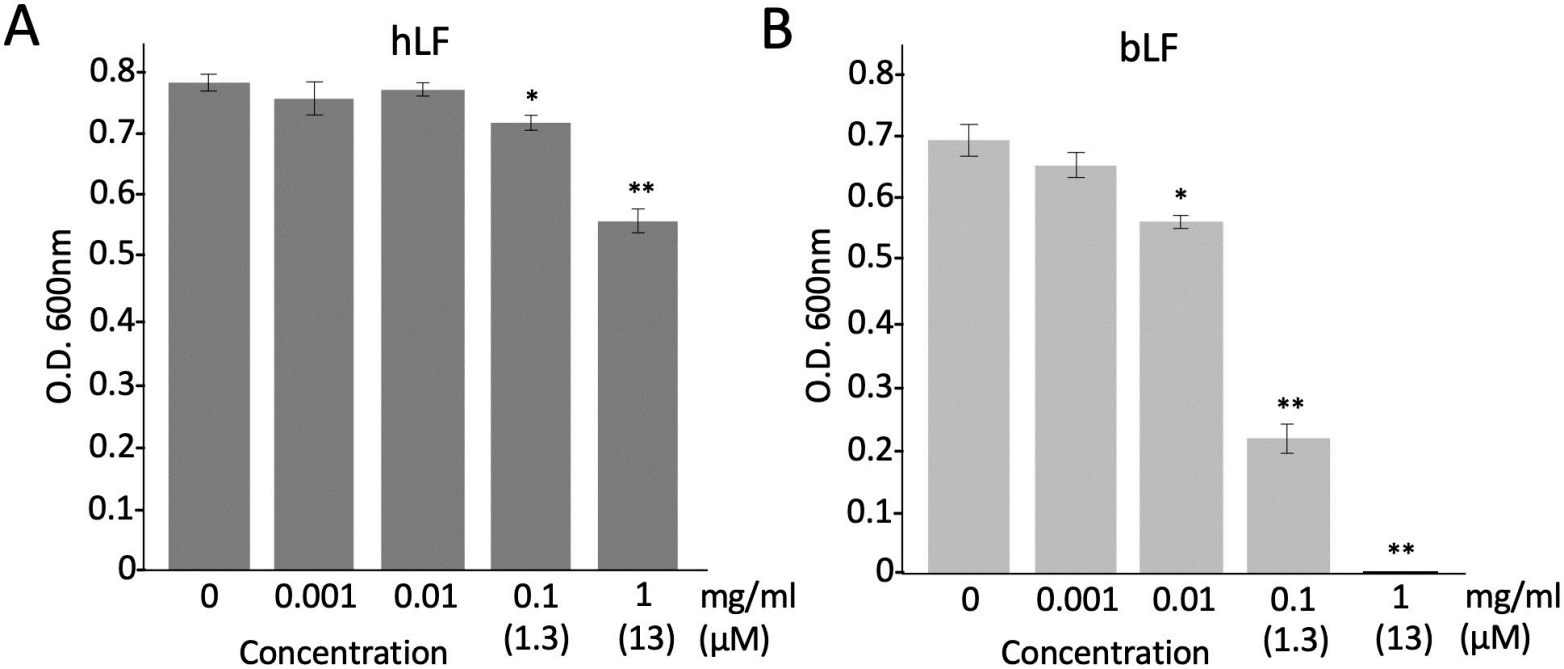
Comparison of the antimicrobial effects of human-derived lactoferrin (hLF) (A) and bovine-derived lactoferrin (bLF) (B) on *Streptomyces scabiei* S58. The mixture of lactoferrin and spores (2 × 10^5^/mL) was incubated at 28 °C for 72 h in tryptic soy broth and the absorbance was measured at 600 nm to monitor bacterial growth. Error bars indicate the mean ± standard division of three independent experiments. Asterisks show a significant difference with respect to the control at *p < 0.05 and **p < 0.01 (Student’s *t* test).

**Fig 2:**
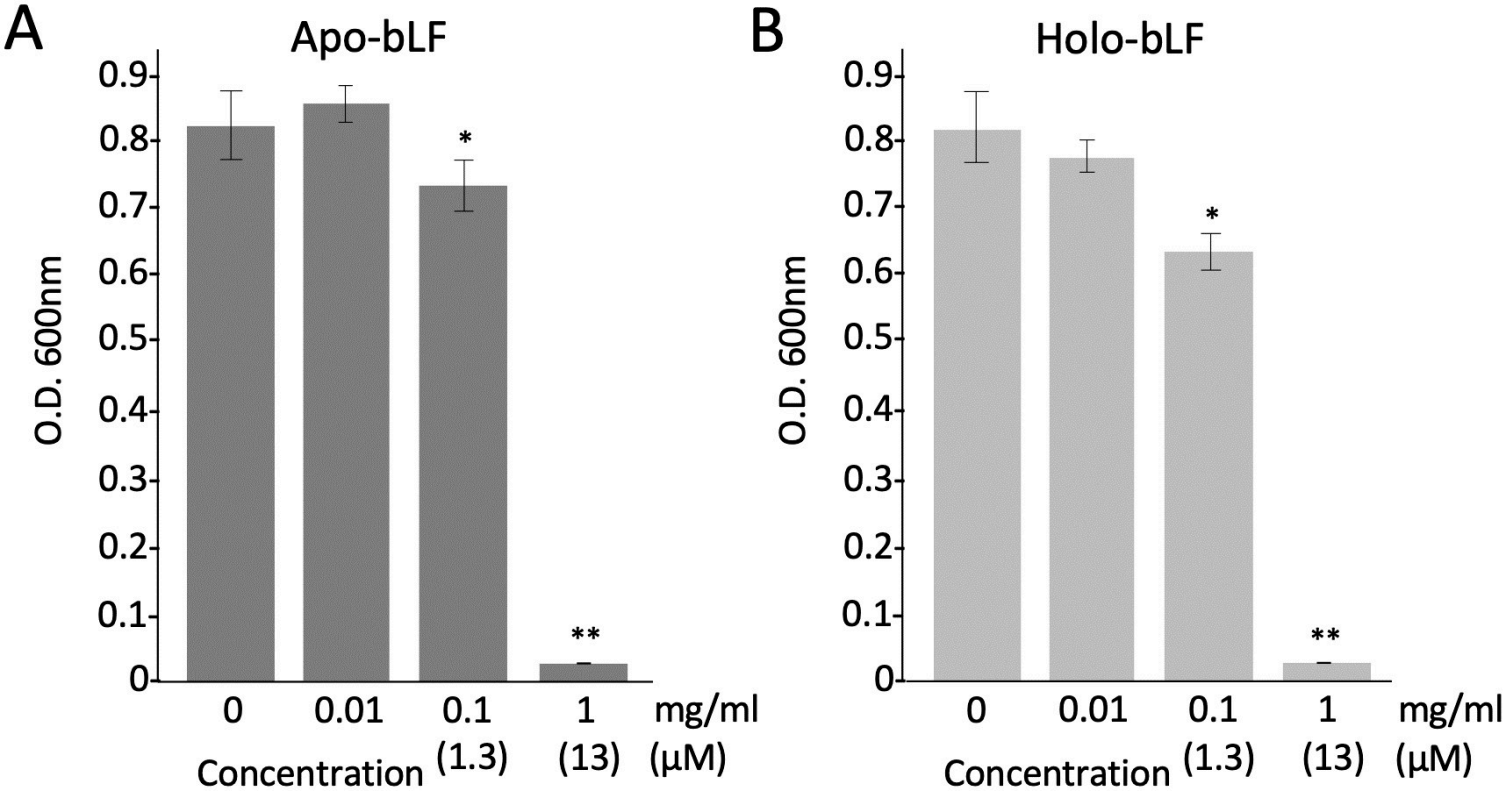
Comparison of the antimicrobial effects of bovine-derived lactoferrin with iron-free (apo-bLF) (A) and bovine-derived lactoferrin saturated with iron (holo-bLF) (B) on *Streptomyces scabiei* S58. The mixture of lactoferrin and spores (2 × 10^5^/mL) was incubated at 28 °C for 72 h in tryptic soy broth and the absorbance was measured at 600 nm to monitor bacterial growth. Error bars indicate the mean ± standard division of three independent experiments. Asterisks show a significant difference with respect to the control at *p < 0.05 and **p < 0.01 (Student’s *t* test).

**Fig 3:**
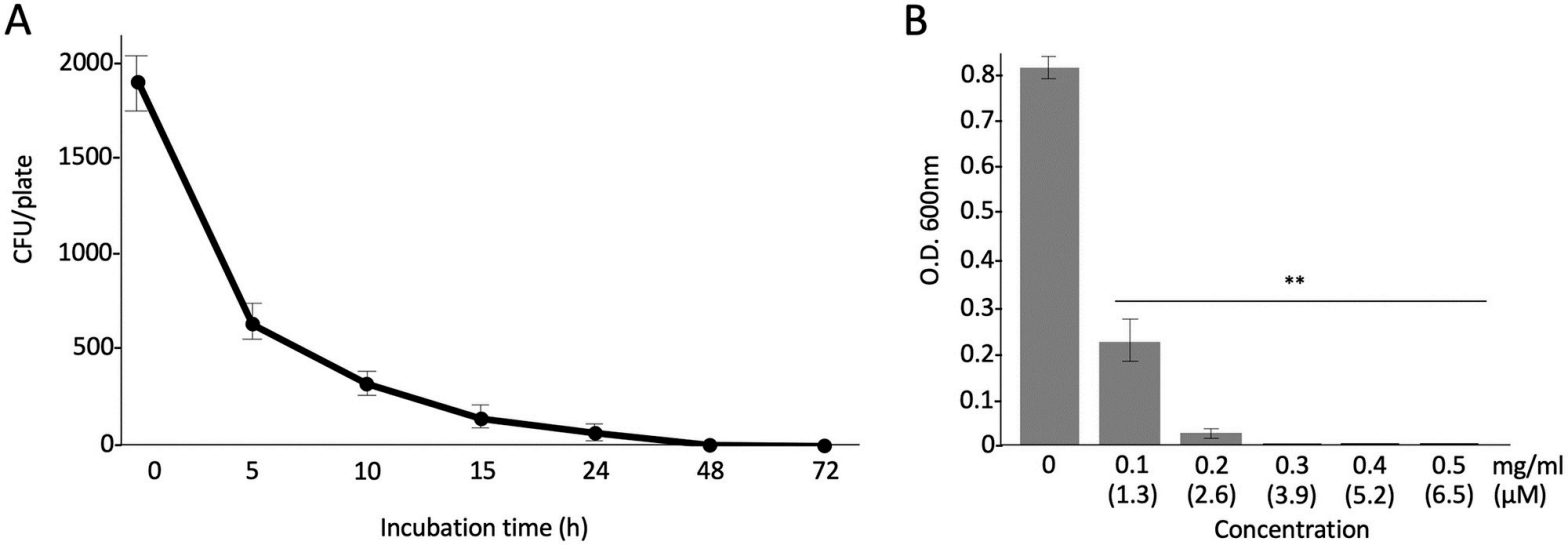
Bactericidal effect of bovine-derived lactoferrin (bLF) (A) and minimal inhibitory concentration (MIC) of bLF (B) on *Streptomyces scabiei* S58. For the assay of bactericidal effect, the mixture of bLF and spores (2 × 10^3^/mL) was incubated at 28 °C and directly cultured on starch agar in a time-dependent manner. The number of colonies emerging on the plates was counted. For the MIC assay, the mixture of bLF and spores (2 × 10^5^/mL) was incubated at 28 °C for 72 h in tryptic soy broth and the absorbance was measured at 600 nm to monitor bacterial growth. Error bars indicate the mean ± standard division of three independent experiments. Asterisks show a significant difference with respect to the control at **p < 0.01 (Student’s *t* test).

We first tested the antimicrobial activities of hLF and bLF on *S*. *scabiei*. The results showed that both hLF and bLF had antimicrobial activity against *S*. *scabiei* and bLF was more potent than hLF (Fig 1). Therefore, we used bLF in further experiments. To understand whether the iron-chelating effect is involved in the antimicrobial action of bLF, we used apo-bLF (iron-free) and holo-bLF (iron-saturated); however, both apo- and holo-bLFs showed the same profiles in their antimicrobial activities (Fig 2), indicating that iron-chelating effect is not involved in its antimicrobial action. Next, we cultured the spores of *S*. *scabiei* on a solid medium in a time-dependent manner after treatment with bLF and counted the number of colonies formed. There was a sharp decrease in the number of colonies at 5 h and only a few colonies remained at 48 h (Fig 3A), indicating that the antimicrobial activity of bLF against *S*. *scabiei* is attributable to its bactericidal effect. The minimal inhibitory concentration of bLF against *S*. *scabiei* was also investigated. No growth of *S*. *scabiei* was observed at a concentration of 3.9 μM and above (Fig 3B).

### Antimicrobial activity of bLF hydrolysate and LFcinB

**Fig 4:**
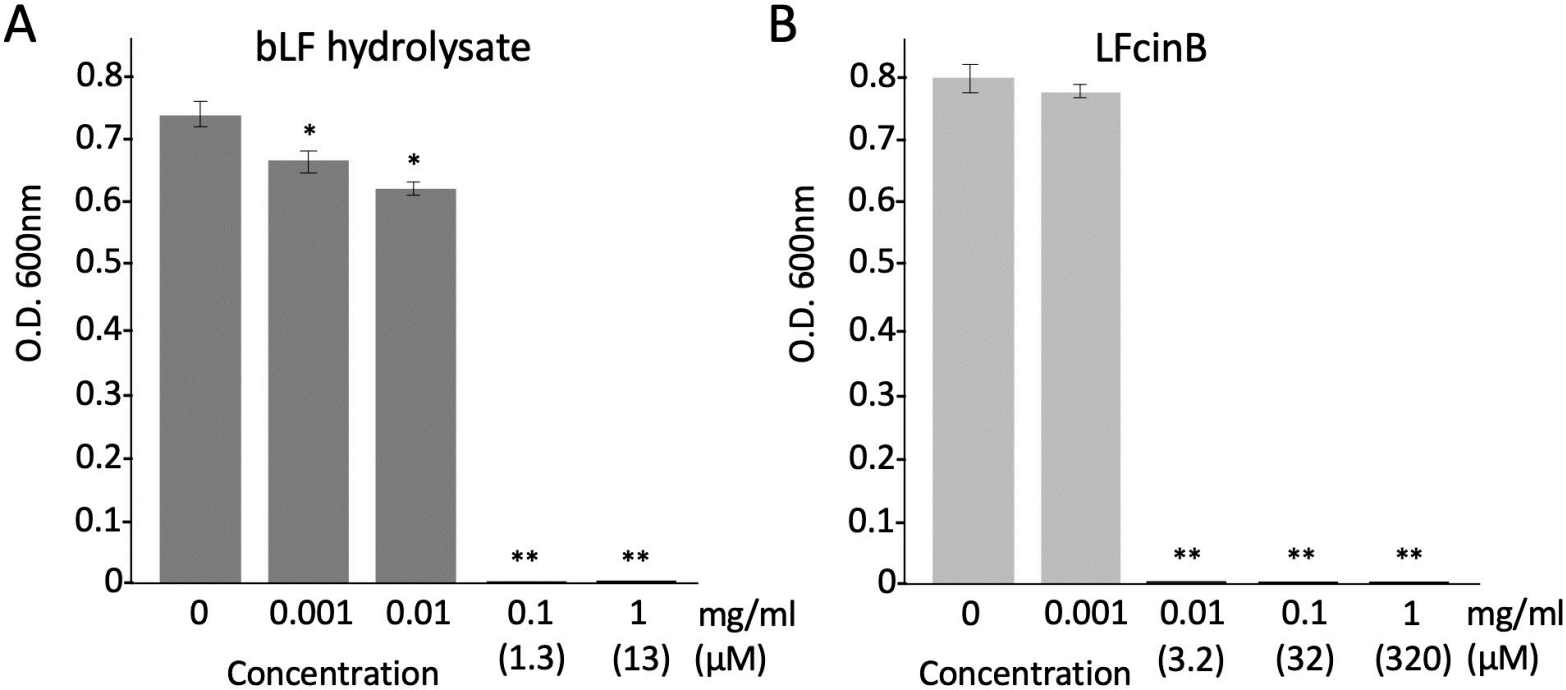
Antimicrobial effects of the hydrolysate of bovine-derived lactoferrin (bLF) digested with porcine pepsin (A) and bovine-derived lactoferrcin (LFcinB) (B). The mixture of each test substance and spores (2 × 10^5^/mL) was incubated at 28 °C for 72 h in tryptic soy broth and the absorbance was measured at 600 nm to monitor bacterial growth. Error bars indicate the mean ± standard division of three independent experiments. Asterisks show a significant difference with respect to the control at *p < 0.05 and **p < 0.01 (Student’s *t* test).

The effects of the hydrolysate of bLF digested with porcine pepsin and LFcinB (25 amino acids), one of the peptides present in the hydrolysate, against *S*. *scabiei* were assessed. The bLF hydrolysate showed a strong activity and *S*. *scabiei* was unable to grow at a concentration of 1.3 μM (Fig 4A). LFcinB was also effective and no growth of *S*. *scabiei* was observed at a concentration of 3.2 μM (Fig 4B). These results indicated that the antimicrobial activity of bLF against *S*. *scabiei* is based on bLF-derived peptides.

### Binding ability of LFcinB to the cell surface

**Fig 5:**
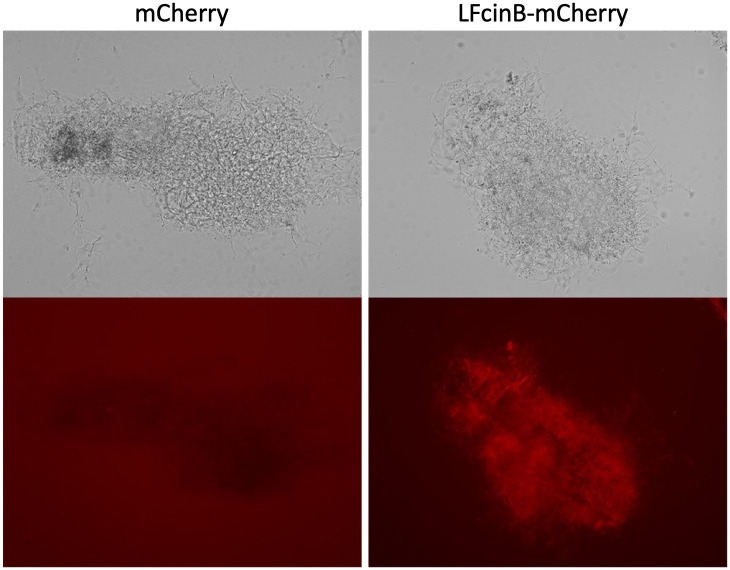
The ability of bovine-derived lactoferrcin (LFcinB) to bind to the cell surface of *Streptomyces scabiei* S58. LFcinB and the fusion protein of mCherry-LFcinB (red fluorescence) were expressed in *Escherichia coli* respectively and adjusted to the concentration of 75 μg/mL before treatment against the pseudohyphal cells of *S*. *scabiei*. The fluorescent image of mCherry-treated cells (lower left) was taken at a longer exposure time (3 s) than that of mCherry-LFcinB-treated cells (1/2 s) (lower right). Upper images were taken in the bright field.

To investigate whether LFcinB binds to the cell surface of *S*. *scabiei*, we constructed a fusion protein of mCherry-LFcinB (S2 Fig). Upon adding the fusion protein to *S*. *scabiei*, the pseudohyphal cells instantly started to glow (Fig 5), and the fluorescence of the cells treated with mCherry alone was not observed even at a longer exposure time (Fig 5, under the left image), indicating that the adhesion of LFcinB to the cell surface is necessary for its lethal effect.

### Antimicrobial activity of synthetic peptides

**Fig 6:**
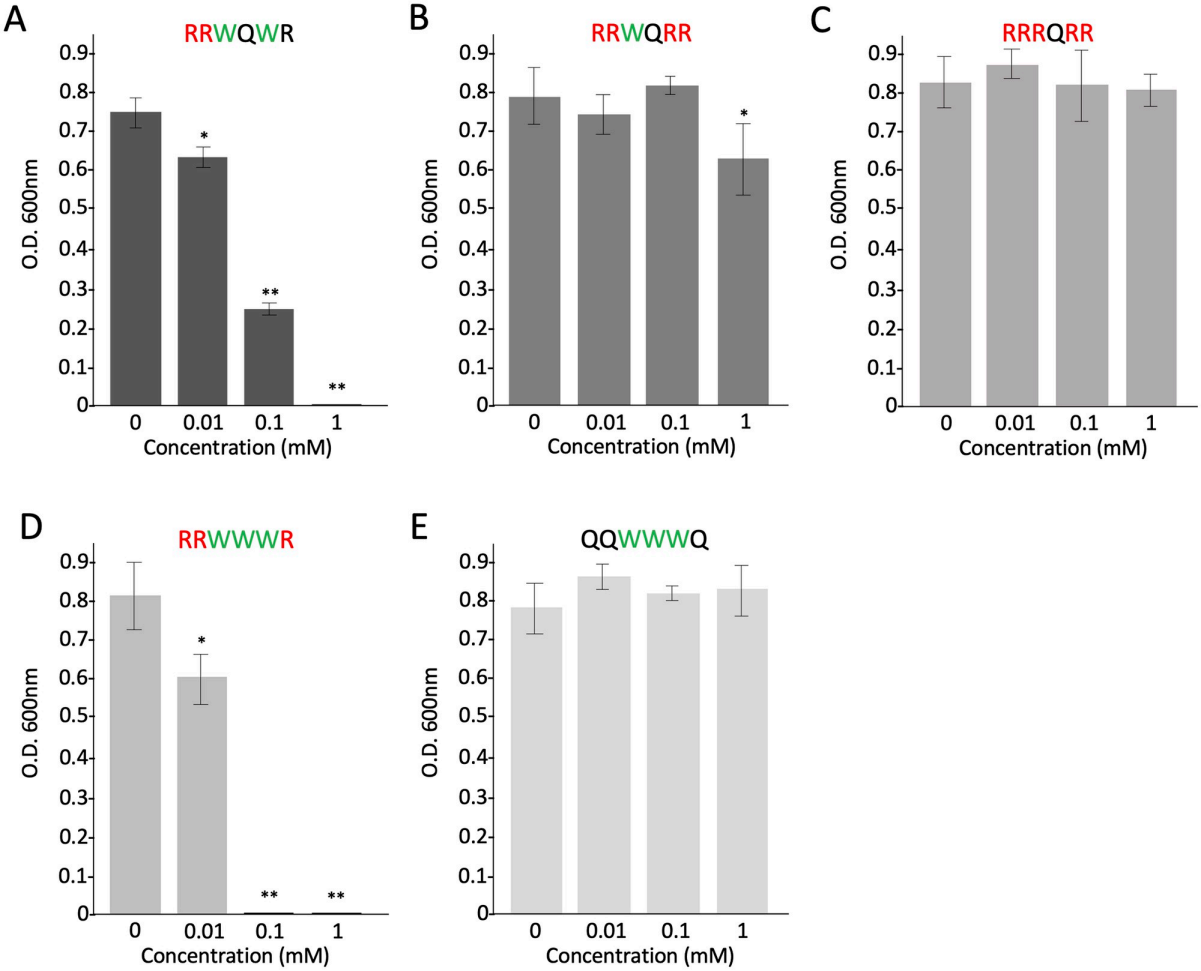
Antimicrobial effects of synthetic peptides on *Streptomyces scabiei* S58. The mixture of each synthetic peptide and spores (2 × 10^5^/mL) was incubated at 28 °C for 72 h in tryptic soy broth and the absorbance was measured at 600 nm to monitor bacterial growth. (A) The original sequence (RRWQWR) situated in bovine-derived lactoferricin. (B) RRWQRR containing only one Trp residue. (C) RRRQRR containing no Trp residue. (D) RRWWWR containing three Trp residues. (E) QQWWWQ containing three Trp residues and no Arg residue. Error bars indicate the mean ± standard division of three independent experiments. Asterisks show a significant difference with respect to the control at *p < 0.05 and **p < 0.01 (Student’s *t* test).

We synthesized the following five peptides: RRWQWR (the original core sequence situated in LFcinB), RRWQRR, RRRQRR, RRWWWR, and QQWWWQ. The antimicrobial effect of each peptide on *S*. *scabiei* is shown in Fig 6. The RRWQRR peptide, containing only one Trp residue, had less activity than the original peptide containing two Trp residues (Fig 6A and 6B). The peptide without Trp residue (RRRQRR) completely lost its activity (Fig 6C). On the contrary, the peptide of RRWWWR containing three Trp residues had the highest activity among the synthetic peptides (Fig 6D). Intriguingly, even if the QQWWWQ peptide contains three Trp residues, in the absence of Arg residue, it caused a complete loss of activity (Fig 6E). These results indicated that Trp residue itself has antimicrobial activity but Arg residue is necessary for its activity at the peptide level.

### Antimicrobial activity of single amino acids (Arg and Trp) and their derivatives

**Fig 7:**
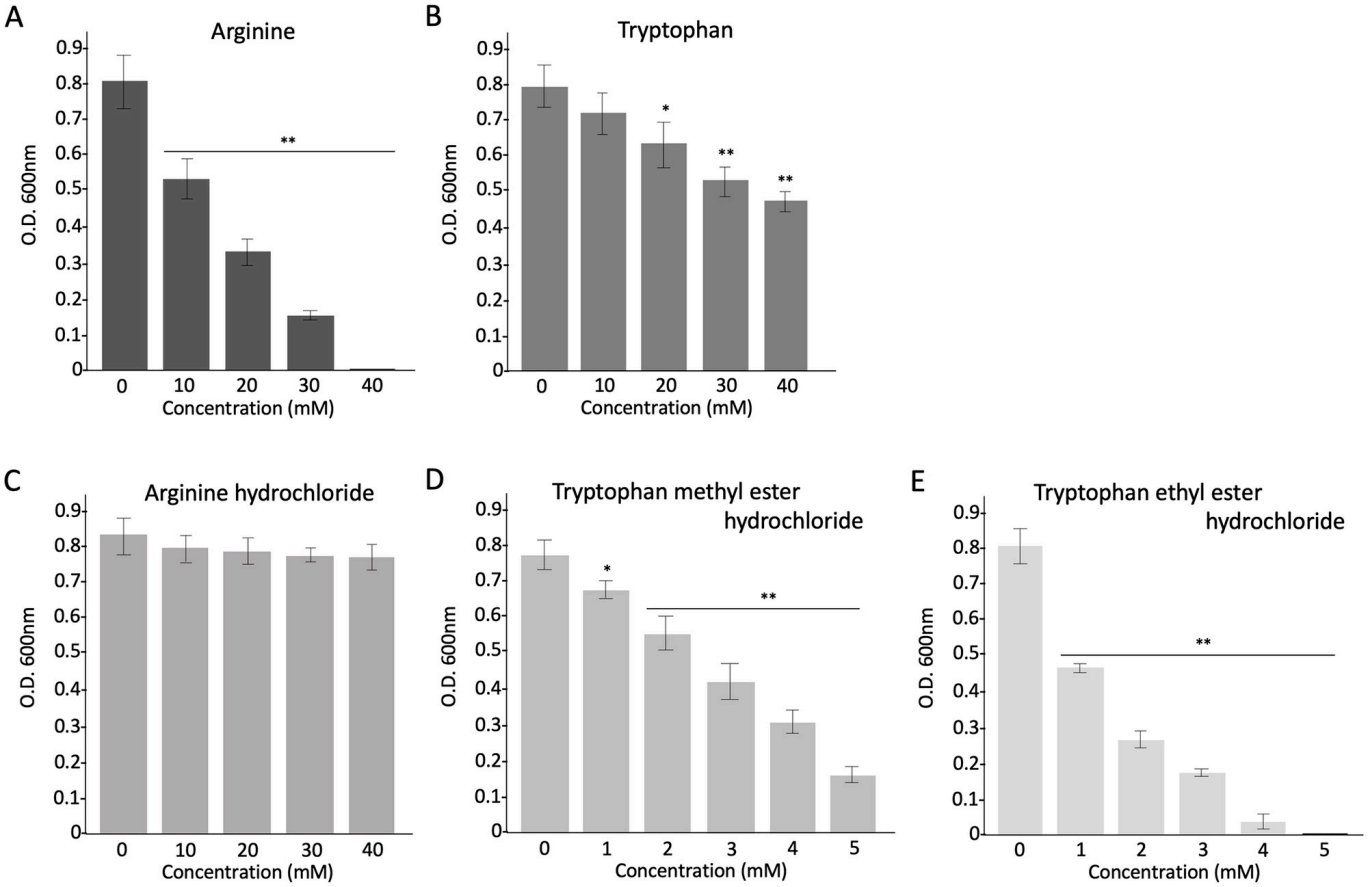
Antimicrobial effects of single amino acids Arg (A) and Trp (B), and their derivatives (C-E) on *Streptomyces scabiei* S58. The mixture of each single amino acid or derivative and spores (2 × 10^5^/mL) was incubated at 28 °C for 72 h in tryptic soy broth and the absorbance was measured at 600 nm to monitor bacterial growth. Error bars indicate the mean ± standard division of three independent experiments. Asterisks show the significant difference with respect to the control at *p < 0.05 and **p < 0.01 (Student’s *t* test).

Unlike the synthetic peptide assay, Arg had a higher activity than that of Trp; however, a high concentration (40 mM) was necessary to completely inhibit the growth of *S*. *scabiei* (Fig 7A and 7B). We also used the derivatives of Arg and Trp to investigate whether the changes in chemical structure affect their antimicrobial activity. As a result, arginine hydrochloride showed a complete loss of activity (Fig 7C), whereas tryptophan methyl ester hydrochloride and tryptophan ethyl ester hydrochloride showed much higher activity than that of tryptophan (Fig 7D and 7E). Tryptophan ethyl ester hydrochloride was the most effective. The assay of these derivatives showed exactly opposite results to those of single amino acids.

## Discussion

The antimicrobial effect of LF on many bacteria, including species pathogenic to humans and plants, has been reported [14–16]. However, the effect of LF on pathogenic actinomycetes has not yet been investigated. This is the first study to report the antimicrobial activity of LF on the potato common scab pathogen *S*. *scabiei* belonging to actinomycetes. The following two main functions are involved in the antimicrobial properties of LF: (i) ability to bind two atoms of ferric iron, leading to the inhibition of bacterial growth via restriction of the availability of iron as a nutrient [24], and (ii) ability to electrostatically bind to the bacterial surface at the peptide level, leading to a direct killing effect by disruption of bacterial membranes [37, 38]. First, we checked whether LF has an antimicrobial effect on *S*. *scabiei* using human and bovine strains and found that LF has antimicrobial activity against *S*. *scabiei* and bLF (bovine) is more effective than hLF (human) (Fig 1). Therefore, we decided to use bLF for further experiments. To clarify whether the iron-chelating property of bLF is involved in its antimicrobial activity (bacteriostatic action) on *S*. *scabiei*, apo-bLF (iron-free) and holo-bLF (iron-saturated) were examined. In some bacterial pathogens, such as *Actinobacillus pleuropneumoniae* and *Cronobacter sakazakii*, iron sequestration by LF inhibits their growth [39, 40]. In this study, the activities of apo- and holo-bLFs against *S*. *scabiei* showed almost the same profiles (Fig 2), indicating that the ability of bLF to bind ferric iron is not responsible for its antimicrobial effect on *S*. *scabiei*. The reason why apo- and holo-bLFs showed lower antimicrobial activity than bLF is that when they were prepared from bLF, their protein structures may have changed slightly, which might have affected their binding ability to the cell surface of *S*. *scabiei*.

Next, to investigate whether its antimicrobial activity is attributable to a direct killing effect, we counted the colony-forming units of *S*. *scabiei* on plates after treatment with bLF in a time-dependent manner. The number of colonies drastically declined at 5 h, and only a few colonies were observed at 48 h (Fig 3A). Thus, bLF has a direct bactericidal effect on *S*. *scabiei*. The bactericidal activity of LF is attributed to its peptides such as LF1-11 [25], lactoferrampin [26], and LFcinB [27]. These three peptides are located in the N1-domain of LF and exhibit a broad antimicrobial action against several gram-negative and gram-positive bacteria, and fungi, including *E*. *coli* [41, 42], *Pseudomonas aeruginosa* [43], *Staphylococcus aureus* [44, 45], *Bacillus subtilis* [46], and *Candida albicans* [47, 48]. Notably, LFcinB, a fragment of 25 residues (LF amino acids 17–41), has a wider spectrum of activity even against viruses [49, 50] and protozoa [51, 52], in addition to microbes. In this study, we used the pepsin hydrolysate of bLF containing fragments of the three peptides and LFcinB alone. Both the hydrolysate and LFcinB were effective against *S*. *scabiei* (Fig 4), indicating that the killing effect of bLF on *S*. *scabiei* is attributable to the bactericidal activity of the peptides. In this study, although we used LFcinB, the other two peptides, LF1-11and lactoferrampin, may also have activities because the bLF hydrolysate had a greater effect than that of bLF (Figs 1B and 4A).

Antimicrobial peptides are rich in cationic and hydrophobic amino acids and are expected to interact with negatively charged elements [53]. Bacterial cell walls have a negative charge; the cell surface components, such as lipopolysaccharides in gram-negative bacteria [54] and teichoic and lipoteichoic acids in gram-positive bacteria, are negatively charged [55]. To test whether LFcinB can bind to the cell surface of *S*. *scabiei*, we expressed an mCherry-LFcinB fusion protein and administered it to the pseudohyphal cells of *S*. *scabiei*. Immediately after adding the fusion protein, the cells started to glow (Fig 5), indicating that LFcinB was electrostatically attracted to the bacterial cell surface.

**Fig 8:**
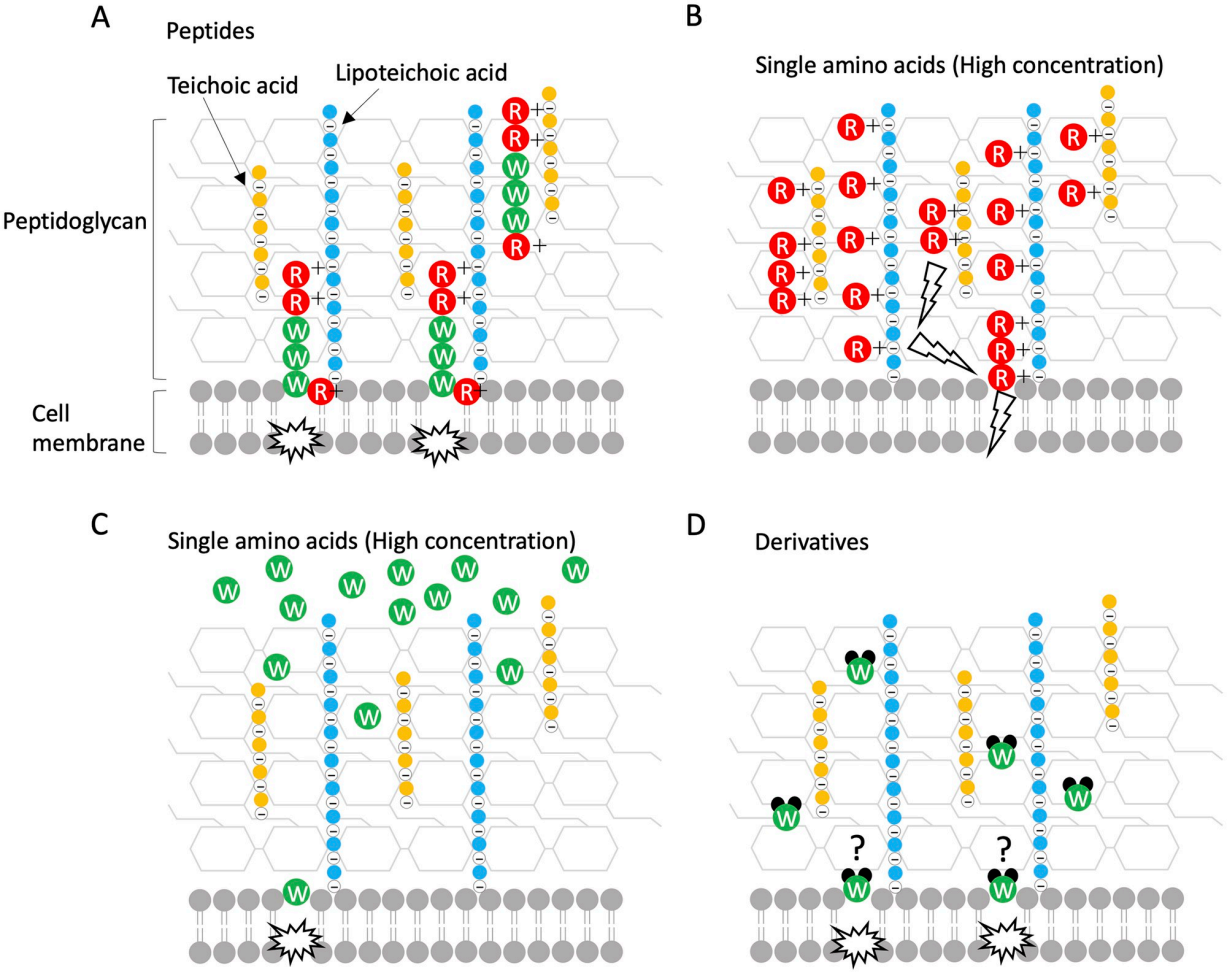
Schematic illustration of the speculated mechanisms for the antimicrobial activities of synthetic peptides, single amino acids, and derivatives against *Streptomyces scabiei* S58. (A) The peptide containing Arg and Trp residues is carried by the cationic Arg residue to the negatively charged bacterial cell surface (teichoic and lipoteichoic acids), and subsequently, Trp residue penetrates the interface layer of the membrane. (B) At a high concentration, cationic Arg may cover the entire negatively charged cell surface and cause an electric potential difference across the membrane, leading to membrane destabilization. (C) Trp, even at a high concentration, cannot effectively reach the cell surface because it is not positively charged. Only a few numbers reach the surface. (D) Trp derivatives may be able to effectively bind to the cell surface without a carrier, such as Arg residues. The change in the chemical structure of Trp may affect its binding ability.

LFcinB contains eight cationic and 11 hydrophobic amino acids out of the 25 residues (S1 Fig). Strøm et al. (2001) reported that two Trp resides in LFcinB cannot be replaced by Ala residue to exert the full antimicrobial activity against *E*. *coli* [56]. The RRWQWR motif containing the two Trp resides in LFcinB is the core site for the antimicrobial activity of the peptide [31–33, 57]. Many studies have reported that positively charged Arg residues first interact with the negatively charged bacterial cell surface, and then hydrophobic Trp residues may provide biological activities using their indole ring, leading to membrane destabilization and subsequent cell lysis [44, 58–61]. Therefore, we synthesized four different peptides by changing the number of Arg and Trp residues in addition to the original motif of RRWQWR. The peptide of RRWWWR containing three Trp residues showed the highest antimicrobial activity against *S*. *scabiei* among the synthetic peptides; the lesser the number of Trp residues, the lower was the effect (Fig 6A, 6B and 6D), and the RRRQRR peptide with no Trp residue completely lost its activity (Fig 6C), indicating that Trp residue is important for the antimicrobial activity against *S*. *scabiei*. In contrast, the QQWWWQ peptide containing three Trp residues but no Arg residue also completely lost its activity, indicating that Arg residue is necessary as well (Fig 6E). In other words, because Trp residue itself cannot bind to the bacterial cell surface, it must be carried by a cationic Arg residue to bind to the negative cell surface (Fig 8A). In the assay performed to compare the antimicrobial activities of hLF and bLF against *S*. *scabiei* (Fig 1), hLF was less effective than bLF, probably because hLF harbors only one Trp residue in its lactoferricin (S1 Fig).

Interestingly, in the antimicrobial assay of single amino acids, Arg showed higher activity than Trp (Fig 7A and 7B); this could be because at a high concentration cationic Arg coated the overall cell surface of *S*. *scabiei* resulting in an electric potential difference across the membrane that leads to membrane destabilization (Fig 8B). On the contrary, in the assay of Trp alone at high concentrations, because Trp cannot bind to the cell surface by itself, only a few numbers could reach the membrane (Fig 8C). In summary, Trp must reach the cell membrane to exert its antimicrobial activity. We speculated that the change in the chemical structures and properties of Arg and Trp may affect their antimicrobial activity, that is, the ability to bind to the bacterial cell surface. Therefore, we used Arg and Trp derivatives, namely arginine hydrochloride, tryptophan methyl ester hydrochloride, and tryptophan ethyl ester hydrochloride, which are easily available. Intriguingly, arginine hydrochloride completely lost the antimicrobial activity against *S*. *scabiei* shown by Arg (Fig 7A and 7C). Sephai et al. (2017) reported that the antimicrobial activity of poly-arginine is influenced by pH; at mild acidic pH (5 and 6), its MIC values decreased [62]. We measured the pH of the medium in which arginine or arginine hydrochloride was dissolved and found it to be 9.5 and 5.5, respectively. *Streptomyces* spp. can grow at pH values ranging from 4.0 to 11.5 [63]. Teichoic and lipoteichoic acids in gram-positive bacterial cell walls are esterified to D-Ala residues and at neutral or lower pH, the amino group of D-Ala (pKa 8.42) is protonated, conferring a neutral or positive charge to the cell walls [64, 65]. Thus, positively charged Arg may have been unable to bind to the cell surface in the medium containing arginine hydrochloride. On the contrary, Trp derivatives showed much higher activity than Trp (Fig 7B, 7D and 7E), indicating that the change in the chemical structures of Trp affects the binding ability to the bacterial cell surface and Trp derivatives can reach the surface by themselves without a carrier (Fig 8D). However, how they reach the cell membrane without the help of a carrier is unclear. As more than 100 commercially available Trp derivatives exist (https://www.chemicalbook.com/ProductCatalog_EN/151727.htm), further effective derivatives need to be investigated. Qin et al. (2020) also reported that indole-based derivatives have great potential as new antimicrobial agents [66]. Amino acid derivatives have the advantage of being inexpensive compared with synthetic peptides, which is useful in reducing the operational costs of a farm. We are currently investigating more effective and safe derivatives to control *S*. *scabiei*. The IC_50_ values of all the test substances used in this study are summarized in Table 1.

**Table 1:** Antimicrobial activity (IC_50_) of test substances used in this study against *Streptomyces scabiei* S58.

Test substances	IC_50_
Proteins (μM)	
Human lactoferrin (hLF)	138.035±7.956
Bovine lactoferrin (bLF)	0.458±0.074
Apo-bLF	2.388±0.203
Holo-bLF	2.177±0.428
Peptides (μM)	
bLF hydrolysate	0.257±0.038
Bovine lactoferricin (LFcinB)	1.222±0.045
Synthetic peptides (mM)	
RRWQWR	0.048±0.009
RRWQRR	25.797±13.034
RRRQRR	N/A
RRWWWR	0.027±0.002
QQWWWQ	N/A
Amino acids (mM)	
Arginine	14.267±1.400
Tryptophan	(40*)
Amino acid derivatives (mM)	
Arginine hydrochloride	N/A
Tryptophan methyl ester hydrochloride	2.859±0.326
Tryptophan ethyl ester hydrochloride	1.303±0.004

The half-maximal inhibitory concentration (IC_50_) values were determined using the probit analysis program. Each value in the table is represented as mean ± standard division (*n* = 3). N/A means that there was no antimicrobial activity.

*The maximum (saturation) concentration of tryptophan was 40mM, which inhibited the growth of *S*. *scabiei* by 40%.

## Supporting information

S1 Fig Protein structure of bovine-derived lactoferrin (bLF) (PDB accession number: 1BLF). The lactoferricin region in bLF (LFcinB) is shown in red. The amino acid sequences of LFcinB and human-derived latoferricin (LFcinH) are shown. The sequence in the box is the core motif of LFcinB and is used for the assay of synthetic peptides.
(TIF)

S2 Fig Fusion protein of mCherry-LFcinB expressed in *Escherichia coli*. Native PAGE was conducted, and the gel was illuminated with UV light so that the fluorescence of mCherry protein could be detected (left). In native PAGE, mCherry-LFcinB (lane 2) appeared as broad bands because LFcinB, which is rich in cationic amino acids, affects the structure and mass-to-charge ratio of the fusion protein. SDS-PAGE was also conducted (middle). It is known that mCherry (DsRed) protein is fragmented at its chromophore group under the conditions of SDS-PAGE (Gross et al. 2000). The largest bands are of uncut proteins (about 30 KDa). Two other bands represent the cleaved fragments. Purified proteins were directly illuminated with UV light (right). Reference: Gross LA, Baird GS, Hoffman RC, Baldridge KK, Tsien RY. The structure of the chromophore within DsRed, a red fluorescent protein from coral. Proc Natl Acad Sci U S A. 2000; 97: 11990–5. 10.1073/pnas.97.22.11990. 11050230; PMCID: PMC17282.
(TIF)

## References

[1] Masson PL, Heremans JF, Dive CH. An iron-binding protein common to many external secretions. Clin Chim Acta 1966; 14: 735–739. 10.1016/0009-8981(66)90004-0

[2] Masson PL, Heremans JF, Schonne E. Lactoferrin, an iron-binding protein in neutrophilic leukocytes. J Exp Med. 1969; 130: 643–658. 10.1084/jem.130.3.643 4979954 PMC2138704

[3] Berlov MN, Korableva ES, Andreeva YV, Ovchinnikova TV, Kokryakov VN. Lactoferrin from canine neutrophils: isolation and physicochemical and antimicrobial properties. Biochemistry. 2007; 72: 445–451. 10.1134/S0006297907040128 17511610

[4] Embleton ND, Berrington JE. Clinical trials of lactoferrin in the newborn: effects on infection and the gut microbiome. Nestle Nutr Inst Workshop Ser. 2020; 94: 141–151. 10.1159/000505334 32160617

[5] Murphy ME, Kariwa H, Mizutani T, Yoshimatsu K, Arikawa J, Takashima I. In vitro antiviral activity of lactoferrin and ribavirin upon hantavirus. Arch Virol. 2000; 145: 1571–1582. 10.1007/s007050070077 11003470

[6] Superti F, Agamennone M, Pietrantoni A, Ammendolia MG. Bovine lactoferrin prevents influenza A virus infection by interfering with the fusogenic function of viral hemagglutinin. Viruses. 2019; 11: 51. 10.3390/v11010051 30641890 PMC6357187

[7] Rogers HJ, Synge C. Bacteriostatic effect of human milk on *Escherichia coli*: the role of IgA. Immunology. 1978; 34: 19–28. 146678 PMC1457336

[8] Ellison RT 3rd, Giehl TJ. Killing of gram-negative bacteria by lactoferrin and lysozyme. J Clin Invest. 1991; 88: 1080–1091. 10.1172/JCI115407 1918365 PMC295557

[9] Cirioni O, Giacometti A, Barchiesi F, Scalise G. Inhibition of growth of *Pneumocystis carinii* by lactoferrins alone and in combination with pyrimethamine, clarithromycin and minocycline. J Antimicrob Chemother. 2000; 46: 577–582. 10.1093/jac/46.4.577 11020255

[10] Kuipers ME, de Vries HG, Eikelboom MC, Meijer DK, Swart PJ. Synergistic fungistatic effects of lactoferrin in combination with antifungal drugs against clinical *Candida* isolates. Antimicrob Agents Chemother. 1999; 43: 2635–2641. 10.1128/AAC.43.11.2635 10543740 PMC89536

[11] Miotto M, Di Rienzo L, Bò L, Boffi A, Ruocco G, Milanetti E. Molecular mechanisms behind anti SARS-CoV-2 action of lactoferrin. Front Mol Biosci. 2021; 8: 607443. 10.3389/fmolb.2021.607443 33659275 PMC7917183

[12] Salaris C, Scarpa M, Elli M, Bertolini A, Guglielmetti S, Pregliasco F, et al. Protective effects of lactoferrin against SARS-CoV-2 infection in vitro. Nutrients. 2021; 13: 328. 10.3390/nu13020328 33498631 PMC7911668

[13] Campione E, Lanna C, Cosio T, Rosa L, Conte MP, Iacovelli F, et al. Lactoferrin as antiviral treatment in COVID-19 management: preliminary evidence. Int J Environ Res Public Health. 2021; 18: 10985. 10.3390/ijerph182010985 34682731 PMC8535893

[14] Han J, Lakshman DK, Galvez LC, Mitra S, Baenziger PS, Mitra A. Transgenic expression of lactoferrin imparts enhanced resistance to head blight of wheat caused by *Fusarium graminearum*. BMC Plant Biol. 2012; 12: 33. 10.1186/1471-2229-12-33 22405032 PMC3364854

[15] Kim WS, Kim PH, Shimazaki K. Sensitivity of *Pseudomonas syringae* to bovine lactoferrin hydrolysates and identification of a novel inhibitory peptide. Korean J Food Sci Anim Resour. 2016; 36: 487–93. 10.5851/kosfa.2016.36.4.487 27621689 PMC5018508

[16] Wang J, Wang HY, Xia XM, Li PP, Wang KY. Inhibitory effect of esterified lactoferin and lactoferrin against tobacco mosaic virus (TMV) in tobacco seedlings. Pestic Biochem Physiol. 2013; 105: 62–68. 10.1016/j.pestbp.2012.11.009 24238292

[17] Liepke C, Adermann K, Raida M, Mägert HJ, Forssmann WG, Zucht HD. Human milk provides peptides highly stimulating the growth of bifidobacteria. Eur J Biochem. 2002; 269: 712–718. 10.1046/j.0014-2956.2001.02712.x 11856332

[18] Goyer C, Beaulieu C. Host range of Streptomycete strains causing common scab. Plant Dis. 1997; 81: 901–904. 10.1094/PDIS.1997.81.8.901 30866378

[19] Loria R, Bukhalid RA, Fry BA, King RR. Plant pathogenicity in the genus *Streptmyces*. Plant Dis. 1997; 81: 836–846. 10.1094/PDIS.1997.81.8.836 30866367

[20] Legrand D, Mazurier J, Colavizza D, Montreuil J, Spik G. Properties of the iron-binding site of the N-terminal lobe of human and bovine lactotransferrins. Importance of the glycan moiety and of the non-covalent interactions between the N- and C-terminal lobes in the stability of the iron-binding site. Biochem J. 1990; 266: 575–581. 2156501 PMC1131170

[21] Kuwata H, Yip TT, Yip CL, Tomita M, Hutchens TW. Bactericidal domain of lactoferrin: detection, quantitation, and characterization of lactoferricin in serum by SELDI affinity mass spectrometry. Biochem Biophys Res Commun. 1998; 245: 764–773. 10.1006/bbrc.1998.8466 9588189

[22] Sijbrandij T, Ligtenberg AJ, Nazmi K, Veerman EC, Bolscher JG, Bikker FJ. Effects of lactoferrin derived peptides on simulants of biological warfare agents. World J Microbiol Biotechnol. 2017; 33: 3. 10.1007/s11274-016-2171-8 27832504 PMC5104768

[23] Cassat JE, Skaar EP. Iron in infection and immunity. Cell Host Microbe. 2013; 13: 509–519. 10.1016/j.chom.2013.04.010 23684303 PMC3676888

[24] Lu J, Francis JD, Guevara MA, Moore RE, Chambers SA, Doster RS, et al. Antibacterial and anti-biofilm activity of the human breast milk glycoprotein lactoferrin against group b *Streptococcus*. Chembiochem. 2021; 22: 2124–2133. 10.1002/cbic.202100016 33755306 PMC8254657

[25] Nibbering PH, Ravensbergen E, Welling MM, van Berkel LA, van Berkel PH, Pauwels EK, et al. Human lactoferrin and peptides derived from its N terminus are highly effective against infections with antibiotic-resistant bacteria. Infect Immun. 2001; 69: 1469–1476. 10.1128/IAI.69.3.1469-1476.2001 11179314 PMC98043

[26] van der Kraan MI, Groenink J, Nazmi K, Veerman EC, Bolscher JG, Nieuw Amerongen AV. Lactoferrampin: a novel antimicrobial peptide in the N1-domain of bovine lactoferrin. Peptides. 2004; 25: 177–183. 10.1016/j.peptides.2003.12.006 15062998

[27] Bellamy W, Takase M, Yamauchi K, Wakabayashi H, Kawase K, Tomita M. Identification of the bactericidal domain of lactoferrin. Biochim Biophys Acta. 1992; 1121: 130–136. doi: 10.1016/0167-4838(92)90346-f 1599934

[28] Groenink J, Walgreen-Weterings E, van ’t Hof W, Veerman EC, Nieuw Amerongen AV. Cationic amphipathic peptides, derived from bovine and human lactoferrins, with antimicrobial activity against oral pathogens. FEMS Microbiol Lett. 1999; 179: 217–222. 10.1111/j.1574-6968.1999.tb08730.x 10518718

[29] Kang JH, Lee MK, Kim KL, Hahm KS. Structure-biological activity relationships of 11-residue highly basic peptide segment of bovine lactoferrin. Int J Pept Protein Res. 1996; 48: 357–363. 10.1111/j.1399-3011.1996.tb00852.x 8919056

[30] Hwang PM, Zhou N, Shan X, Arrowsmith CH, Vogel HJ. Three-dimensional solution structure of lactoferricin B, an antimicrobial peptide derived from bovine lactoferrin. Biochemistry. 1998; 37: 4288–4298. 10.1021/bi972323m 9521752

[31] Vega SC, Martínez DA, Chalá MDS, Vargas HA, Rosas JE. Design, synthesis and evaluation of branched RRWQWR-based peptides as antibacterial agents against clinically relevant gram-positive and gram-negative pathogens. 2018; 9:329. doi: 10.3389/fmicb.2018.00329 29551999 PMC5840262

[32] Ueta E, Tanida T, Osaki T. A novel bovine lactoferrin peptide, FKCRRWQWRM, suppresses *Candida* cell growth and activates neutrophils. J Pept Res. 2001; 57: 240–9. doi: 10.1111/j.1399-3011.2001.00821.x 11298926

[33] Hossain F, Dohra H, Yamazaki M. Effect of membrane potential on entry of lactoferricin B-derived 6-residue antimicrobial peptide into single *Escherichia coli* cells and lipid vesicles. J Bacteriol. 2021; 203: e00021–21. doi: 10.1128/JB.00021-21 33558393 PMC8092161

[34] Nishi Y, Takeuchi T, Suzuki F, Suga Y, Tashiro N, Nakamura M, et al. Distribution of species in *Streptomyces* isolates causing potato common scab in Kagoshima and Nagasaki prefectures. Jpn J Phytopathol. 2015; 81: 22–31. 10.3186/jjphytopath.81.22

[35] Wang XY, Guo HY, Zhang W, Wen PC, Zhang H, Guo ZR, et al. Effect of iron saturation level of lactoferrin on osteogenic activity in vitro and in vivo. J Dairy Sci. 2013; 96: 33–39. 10.3168/jds.2012-5692 23164231

[36] Tomita M, Bellamy W, Takase M, Yamauchi K, Wakabayashi H, Kawase K. Potent antibacterial peptides generated by pepsin digestion of bovine lactoferrin. J Dairy Sci. 1991; 74: 4137–4142. doi: 10.3168/jds.S0022-0302(91)78608-6 1787185

[37] Orsi N. The antimicrobial activity of lactoferrin: current status and perspectives. Biometals. 2004; 17: 189–196. 10.1023/b:biom.0000027691.86757.e2 15222464

[38] Látrová K, Havlová N, Večeřová R, Pinkas D, Bogdanová K, Kolář M, et al. Outer membrane and phospholipid composition of the target membrane affect the antimicrobial potential of first- and second-generation lipophosphonoxins. Sci Rep. 2021; 11: 10446. 10.1038/s41598-021-89883-0 34001940 PMC8129119

[39] Luna-Castro S, Aguilar-Romero F, Samaniego-Barrón L, Godínez-Vargas D, de la Garza M. Effect of bovine apo-lactoferrin on the growth and virulence of *Actinobacillus pleuropneumoniae*. Biometals. 2014; 27: 891–903. 10.1007/s10534-014-9752-5 24878848

[40] Harouna S, Carraminana JJ, Navarro F, Perez MD, Calvo M, et al. Antibacterial activity of bovine milk lactoferrin on the emerging foodborne pathogen *Cronobacter sakazakii*: effect of media and heat treatment. Food Control 47: 520–525. 10.1016/j.foodcont.2014.07.061

[41] Flores-Villaseñor H, Canizalez-Román A, Reyes-Lopez M, Nazmi K, de la Garza M, Zazueta-Beltrán J, et al. Bactericidal effect of bovine lactoferrin, LFcin, LFampin and LFchimera on antibiotic-resistant *Staphylococcus aureus* and *Escherichia coli*. Biometals. 2010; 23: 569–578. 10.1007/s10534-010-9306-4 20195887

[42] van der Kraan MI, van Marle J, Nazmi K, Groenink J, van ’t Hof W, Veerman EC, et al. Ultrastructural effects of antimicrobial peptides from bovine lactoferrin on the membranes of *Candida albicans* and *Escherichia coli*. Peptides. 2005; 26: 1537–1542. doi: 10.1016/j.peptides.2005.02.011 16112390

[43] Ramamourthy G, Vogel HJ. Antibiofilm activity of lactoferrin-derived synthetic peptides against *Pseudomonas aeruginosa* PAO1. Biochem Cell Biol. 2021; 99: 138–148. 10.1139/bcb-2020-0253 32871093

[44] Huertas NJ, Monroy ZJR, Medina RF, Castañeda JEG. Antimicrobial activity of truncated and polyvalent peptides derived from the FKCRRQWQWRMKKGLA sequence against *Escherichia coli* ATCC 25922 and *Staphylococcus aureus* ATCC 25923. Molecules. 2017; 22: 987. 10.3390/molecules22060987 28613262 PMC6152618

[45] Bai X, Teng D, Tian Z, Zhu Y, Yang Y, Wang J. Contribution of bovine lactoferrin inter-lobe region to iron binding stability and antimicrobial activity against *Staphylococcus aureus*. Biometals. 2010; 23: 431–439. 10.1007/s10534-010-9300-x 20145976

[46] Ulvatne H, Samuelsen Ø, Haukland HH, Krämer M, Vorland LH. Lactoferricin B inhibits bacterial macromolecular synthesis in *Escherichia coli* and *Bacillus subtilis*. FEMS Microbiol Lett. 2004; 237: 377–384. 10.1016/j.femsle.2004.07.001 15321686

[47] Bellamy W, Wakabayashi H, Takase M, Kawase K, Shimamura S, Tomita M. Killing of *Candida albicans* by lactoferricin B, a potent antimicrobial peptide derived from the N-terminal region of bovine lactoferrin. Med Microbiol Immunol. 1993; 182: 97–105. 10.1007/BF00189377 8332105

[48] Chang CK, Kao MC, Lan CY. Antimicrobial activity of the peptide LfcinB15 against *Candida albicans*. J Fungi (Basel). 2021; 7: 519. 10.3390/jof7070519 34209722 PMC8306953

[49] Pietrantoni A, Ammendolia MG, Tinari A, Siciliano R, Valenti P, Superti F. Bovine lactoferrin peptidic fragments involved in inhibition of Echovirus 6 in vitro infection. Antiviral Res. 2006; 69: 98–106. 10.1016/j.antiviral.2005.10.006 16386316

[50] Shestakov A, Jenssen H, Nordström I, Eriksson K. Lactoferricin but not lactoferrin inhibit herpes simplex virus type 2 infection in mice. Antiviral Res. 2012; 93: 340–345. 10.1016/j.antiviral.2012.01.003 22269645

[51] Frontera LS, Moyano S, Quassollo G, Lanfredi-Rangel A, Rópolo AS, Touz MC. Lactoferrin and lactoferricin endocytosis halt *Giardia* cell growth and prevent infective cyst production. Sci Rep. 2018; 8: 18020. 10.1038/s41598-018-36563-1 30575774 PMC6303297

[52] Omata Y, Satake M, Maeda R, Saito A, Shimazaki K, Yamauchi K, et al. Reduction of the infectivity of *Toxoplasma gondii* and *Eimeria stiedai* sporozoites by treatment with bovine lactoferricin. J Vet Med Sci. 2001; 63: 187–190. 10.1292/jvms.63.187 11258458

[53] Bechinger B, Gorr SU. Antimicrobial peptides: mechanisms of action and resistance. J Dent Res. 2017; 96: 254–260. 10.1177/0022034516679973 27872334 PMC5298395

[54] Schwechheimer C, Kuehn MJ. Outer-membrane vesicles from Gram-negative bacteria: biogenesis and functions. Nat Rev Microbiol. 2015; 13: 605–619. 10.1038/nrmicro3525 26373371 PMC5308417

[55] Brown S, Santa Maria JP Jr, Walker S. Wall teichoic acids of gram-positive bacteria. Annu Rev Microbiol. 2013; 67: 313–336. 10.1146/annurev-micro-092412-155620 24024634 PMC3883102

[56] Strøm MB, Rekdal O, Svendsen JS. Antibacterial activity of 15-residues lactoferricin derivatives. J Pept Res. 2000; 56:265–274. doi: 10.1034/j.1399-3011.2000.00770.x 11095180

[57] Tomita M, Takase M, Bellamy W, Shimamura S. A review: the active peptide of lactoferrin. Acta Paediatr Jpn. 1994; 36: 585–591. 10.1111/j.1442-200x.1994.tb03250.x 7825467

[58] Bocchinfuso G, Palleschi A, Orioni B, Grande G, Formaggio F, Toniolo C, et al. Different mechanisms of action of antimicrobial peptides: insights from fluorescence spectroscopy experiments and molecular dynamics simulations. J Pept Sci. 2009; 15: 550–558. 10.1002/psc.1144 19455510

[59] Farnaud S, Evans RW. Lactoferrin a multifunctional protein with antimicrobial properties. Mol Immunol. 2003; 40: 395–405. doi: 10.1016/s0161-5890(03)00152-4 14568385

[60] Umeyama M, Kira A, Nishimura K, Naito A. Interactions of bovine lactoferricin with acidic phospholipid bilayers and its antimicrobial activity as studied by solid-state NMR. Biochim Biophys Acta. 2006; 1758: 1523–1528. 10.1016/j.bbamem.2006.06.014 16884683

[61] Romo TD, Bradney LA, Greathouse DV, Grossfield A. Membrane binding of an acyl-lactoferricin B antimicrobial peptide from solid-state NMR experiments and molecular dynamics simulations. Biochim Biophys Acta. 2011; 1808: 2019–2030. 10.1016/j.bbamem.2011.03.017 21477580 PMC3124939

[62] Sepahi M, Jalal R, Mashreghi M. Antibacterial activity of poly-l-arginine under different conditions. Iran J Microbiol. 2017; 9: 103–111. 29214002 PMC5715275

[63] Kontro M, Lignell U, Hirvonen MR, Nevalainen A. pH effects on 10 *Streptomyces* spp. growth and sporulation depend on nutrients. Lett Appl Microbiol. 2005; 41: 32–8. doi: 10.1111/j.1472-765X.2005.01727.x . 15960749

[64] Neuhaus FC, Baddiley J. A continuum of anionic charge: structures and functions of D-alanyl-teichoic acids in gram-positive bacteria. Microbiol Mol Biol Rev. 2003; 67: 686–723. doi: 10.1128/MMBR.67.4.686-723.2003 14665680 PMC309049

[65] Bernal P, Zloh M, Taylor PW. Disruption of D-alanyl esterification of *Staphylococcus aureus* cell wall teichoic acid by β-lactam resistance modifier (-)-epicatechin gallate. J Antimicrob Chemother. 2009; 63:1156–1162. doi: 10.1093/jac/dkp094 19307172 PMC2680342

[66] Qin HL, Liu J, Fang WY, Ravindar L, Rakesh KP. Indole-based derivatives as potential antibacterial activity against methicillin-resistance *Staphylococcus aureus* (MRSA). Eur J Med Chem. 2020; 194: 112245. 10.1016/j.ejmech.2020.112245 . 32220687

