# Supplementary figures and images for: Antimicrobial effect and mechanism of bovine lactoferrin against the potato common scab pathogen Streptomyces scabiei
Source: PLoS One. 2022 Feb 25;17(2):e0264094. doi: 10.1371/journal.pone.0264094 (PMC8880714; doi:10.1371/journal.pone.0264094)

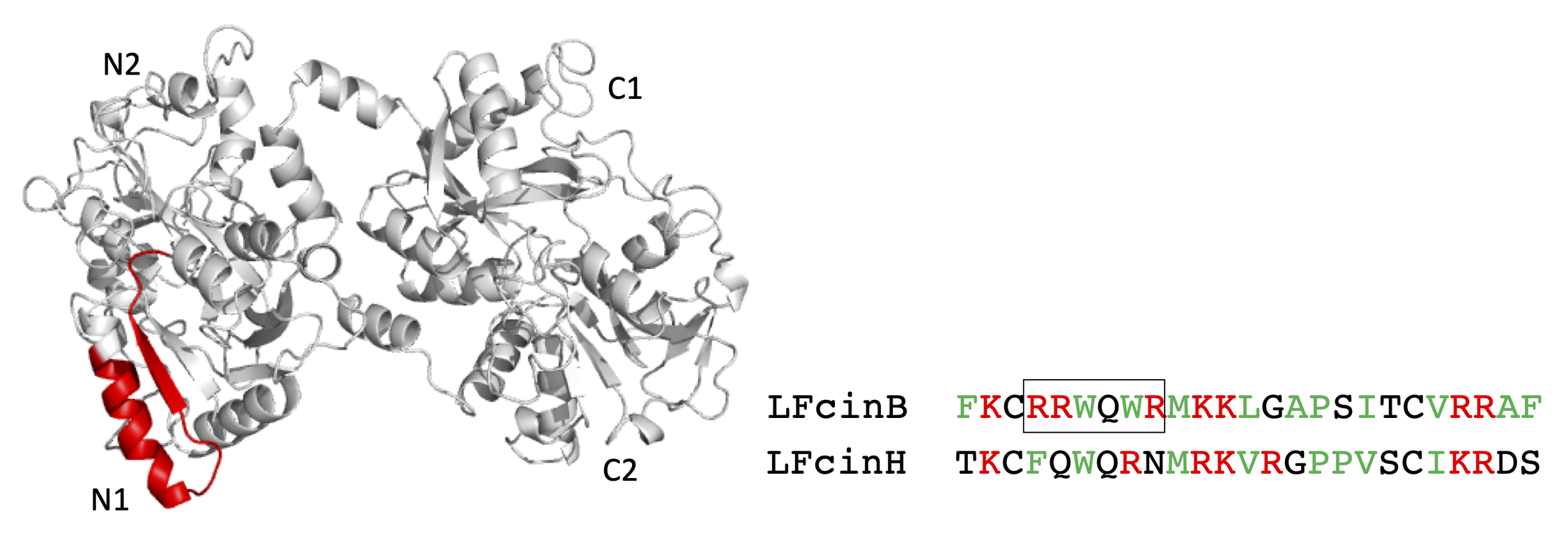

Supplement: S1 Fig — The lactoferricin region in bLF (LFcinB) is shown in red. The amino acid sequences of LFcinB and human-derived latoferricin (LFcinH) are shown. The sequence in the box is the core motif of LFcinB and is used for the assay of synthetic peptides. (TIF) [file pone.0264094.s001.tif]

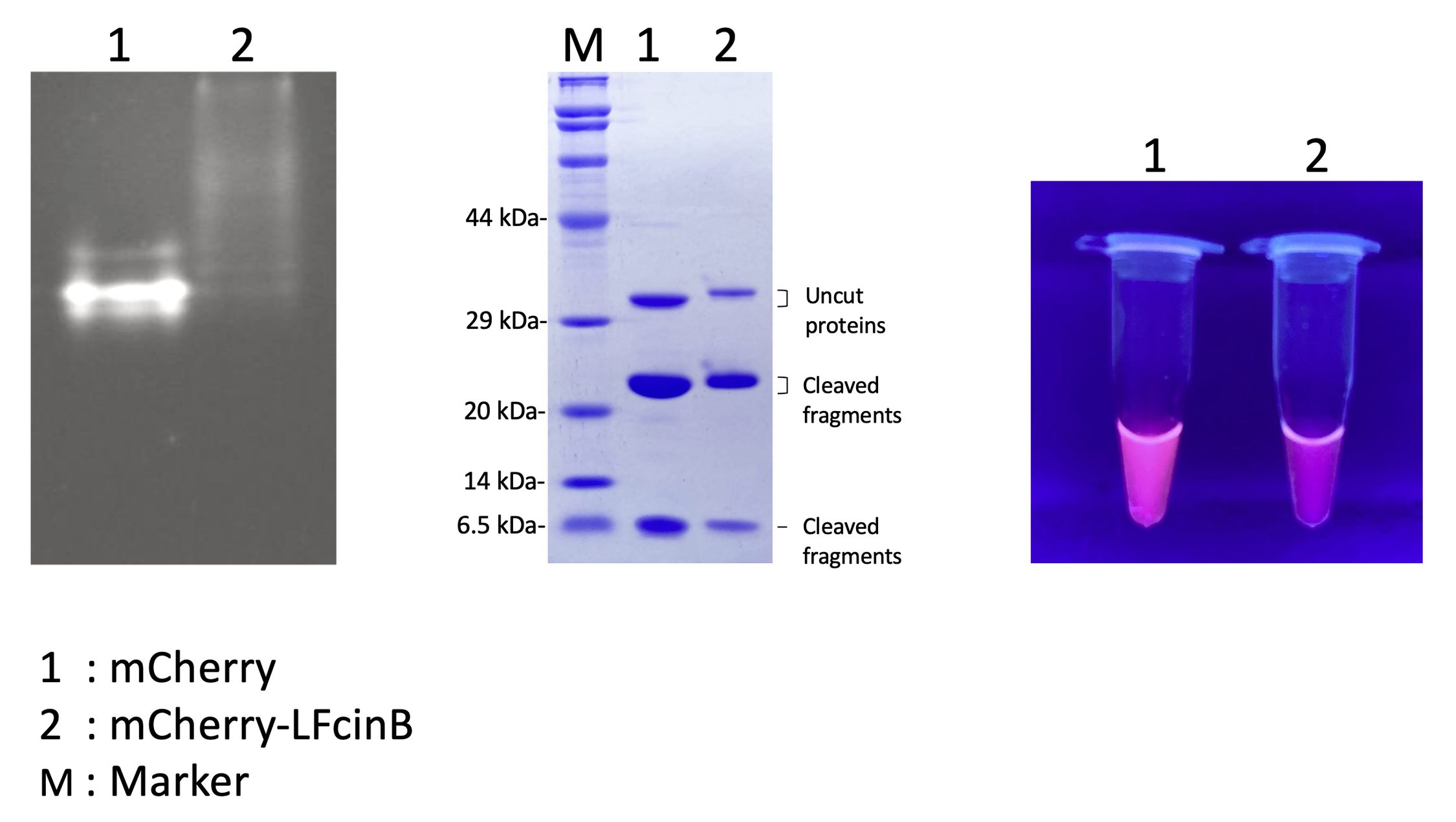

Supplement: S2 Fig — Native PAGE was conducted, and the gel was illuminated with UV light so that the fluorescence of mCherry protein could be detected (left). In native PAGE, mCherry-LFcinB (lane 2) appeared as broad bands because LFcinB, which is rich in cationic amino acids, affects the structure and mass-to-charge ratio of the fusion protein. SDS-PAGE was also conducted (middle). It is known that mCherry (DsRed) protein is fragmented at its chromophore group under the conditions of SDS-PAGE (Gross et al. 2000). The largest bands are of uncut proteins (about 30 KDa). Two other bands represent the cleaved fragments. Purified proteins were directly illuminated with UV light (right). Reference: Gross LA, Baird GS, Hoffman RC, Baldridge KK, Tsien RY. The structure of the chromophore within DsRed, a red fluorescent protein from coral. Proc Natl Acad Sci U S A. 2000; 97: 11990–5. 10.1073/pnas.97.22.11990. 11050230; PMCID: PMC17282. (TIF) [file pone.0264094.s002.tif]
